# Relationships of circulating CD4^+^ T cell subsets and cytokines with the risk of relapse in patients with Crohn’s disease

**DOI:** 10.3389/fimmu.2022.864353

**Published:** 2022-11-04

**Authors:** Rémi Duclaux-Loras, Gilles Boschetti, Bernard Flourie, Xavier Roblin, Jean-Benoit Leluduec, Stéphane Paul, Thibaut Almeras, Karine Ruel, Anthony Buisson, Jacques Bienvenu, Cendrine Josson, Renaud Jasnowski, Stéphane Legastelois, Arnaud Foussat, Camille Meunier, Christophe Viret, Aurore Rozieres, Mathias Faure, Dominique Kaiserlian, Stéphane Nancey

**Affiliations:** ^1^ Centre International de Recherche en Infectiologie (CIRI), Univ Lyon, Institut national de la santé et de la recherche médical (Inserm), U1111, Université Claude Bernard Lyon 1, Centre National de Recherche Scientifique (CNRS), UMR5308, Ecole National Supérieur (ENS) de Lyon, Lyon, France; ^2^ Department of Gastroenterology, Lyon-Sud Hospital, University Claude Bernard Lyon 1 and Hospices Civils de Lyon, Pierre-Bénite, France; ^3^ Department of Gastroenterology, University Hospital, Saint-Etienne, France; ^4^ Laboratory of Immunology and Immunomonitoring, University Hospital, Université de Lyon, Saint-, Etienne, France; ^5^ Department of Gastroenterology, University Hospital, Clermont-Ferrand, France; ^6^ Laboratory of Immunology, Hospices Civils de Lyon, Hopital Lyon-Sud, Pierre-Bénite, France; ^7^ Advanced Bioscience Laboratories (ABL), Lyon, France; ^8^ Indicia, Lyon, France; ^9^ TxCell, Sophia-Antipolis, France

**Keywords:** Crohn’s disease, CD4^+^ T cells, FOXP3, interleukin 17A, relapse

## Abstract

**Background and aims:**

We aimed to analyze circulating CD4^+^ T cell subsets and cytokines during the course of Crohn’s disease (CD).

**Methods and results:**

CD4^+^ T cell subsets, ultrasensitive C-reactive protein (usCRP), and various serum cytokines (IL-6, IL-8, IL-10, IL-13, IL-17A, IL-23, TNFα, IFNγ, and TGFβ) were prospectively monitored every 3 months for 1 year, using multicolor flow cytometry and an ultrasensitive Erenna method in CD patients in remission at inclusion. Relapse occurred in 35 out of the 113 consecutive patients (31%). For patients in remission within 4 months prior to relapse and at the time of relapse, there was no significant difference in Th1, Th17, Treg, and double-positive CD4^+^ T cell subsets co-expressing either IFNγ and FOXP3, IL-17A and FOXP3, or IFNγ and IL-17A. On the contrary, in patients who remained in remission, the mean frequency and number of double-positive IL-17A^+^FOXP3^+^ CD4^+^ T cells and the level of usCRP were significantly higher (*p* ≤ 0.01) 1 to 4 months prior to relapse. At the time of relapse, only the IL-6 and usCRP levels were significantly higher (*p* ≤ 0.001) compared with those patients in remission. On multivariate analysis, a high number of double-positive IL-17A^+^FOXP3^+^ CD4^+^ T cells (≥1.4 cells/mm3) and elevated serum usCRP (≥3.44 mg/L) were two independent factors associated with risk of relapse.

**Conclusions:**

Detection of circulating double-positive FOXP3^+^IL-17A^+^ CD4^+^ T cell subsets supports that T cell plasticity may reflect the inflammatory context of Crohn’s disease. Whether this subset contributes to the pathogenesis of CD relapse needs further studies.

## Introduction

Crohn’s disease (CD) is a chronic relapsing inflammatory disorder characterized by an alternance of flare and remission periods. The occurrence of clinical relapse in CD patients in medically induced remission is a frequent event (30–50% in 1 year) that remains largely unpredictable (1, 2). Identification of novel accurate markers capable of predicting the risk of relapse remains an unmet need in inflammatory bowel diseases (IBD).

CD pathogenesis results from an aberrant mucosal immune response to gut microbiota occurring under genetic predispositions (3). Several studies in mice have highlighted a clear mucosal immunologic imbalance between effector and regulator T cell subsets that drive gut inflammation and lesions (4). Naïve CD4^+^ T helper (Th) cells that encounter their specific antigens presented *via* the major histocompatibility complex molecules by professional antigen-presenting cells differentiate into effector cells which are able to produce various cytokines and possess immunoregulatory/suppressive functions. Apart from the Th1 and Th2 CD4^+^ effector subsets described in 1986 by Mosmann et al. (5), a more recent effector CD4^+^ T cell subset (Th17) producing IL-17A infiltrates mucosal tissues, thus contributing to gut inflammation and damages (6). The differentiation of IL-17A-producing CD4^+^ T cell subpopulations is dependent on the transcription factor RORγt and the presence of various cytokines, most notably TGFβ, within the microenvironment (7). The regulatory T cells (Tregs) characterized by the expression of the transcription factor forkhead box P3 (FOXP3) has been recognized as the most widely studied CD4^+^ T cell immunoregulatory subset promoting tissue homeostasis (8). These Tregs infiltrate the *lamina propria* of the digestive tract where they modulate immune responses to commensal microbes by suppressing the activation, proliferation, and effector functions of adaptive immune cells but are also present in the circulating compartment. Similarly to other T cells, it is now well recognized that they encompass two distinct entities, both in mouse and human, including natural [*i*.*e*., thymus-derived Treg originating from the thymus (tTregs)] and induced peripheral Treg that derive from the neoconversion of T cells in the periphery (pTregs) depending on the environmental conditions, especially in the gut, as demonstrated for “naïve” CD4^+^ T cells upon exposure to TGFβ (9) *in vitro* or in mouse models of colitis as we recently demonstrated *in vivo* using Foxp3 transgenic reporter mice and dextran sulfate colitis or CD4^+^ T cell models (10). In addition, Tregs were shown to acquire the capacity to express either IL-17A or IFNγ under various conditions of *in vitro* stimulation (11, 12). Interestingly, IL-17A-producing cells sharing Treg signatures have been reported to be present in higher frequencies in active CD patients than in healthy volunteers (12). Although the complexity of the functional phenotype of TH17 cells is still incompletely characterized in human, in part due to their plasticity to environmental stimuli and threats from the inflammatory milieu, they are believed to encompass cells producing mediators, including IL17 and other soluble factors, which seem to play an essential role in the maintenance or acquisition of gut homeostasis. Indeed Th17 cells express IL-22, an immune regulatory cytokine mandatory for epithelial barrier functions. Moreover, IL-17A is important for the induction of antimicrobial peptides, such as defensins, which are released rapidly in the epithelial crypts following microbiota insults to protect from epithelial barrier permeability. Thus, compelling experimental evidence in both mouse and human studies supports a potential contribution of Treg/Th17 cells in IBD pathogenesis and highlights that T cell plasticity may underlie the occurrence of flares in IBD (11–13).

Data on the frequencies of these novel circulating double-positive T cell subsets are scarce in patients with CD, leaving the potential impact of their dynamics on the disease uncharacterized. In the present study, we followed a cohort of CD patients in remission every 3 months for 1 year (or less in case of disease relapse) in order to (i) analyze the frequencies and numbers of circulating Th1, Th17, Tregs, and double-positive T cell subsets along with a wide panel of serum cytokines by an ultrasensitive method and (ii) assess the relationships of these immunological parameters with the risk of disease relapse.

## Patients and methods

### Study design

All consecutive outpatients (≥18 years old) with a diagnosis of CD dating for at least 6 months were screened for eligibility between November 2013 and October 2015 in our tertiary referral center in IBD. They were included if they were in medically induced clinical remission for at least three months, defined by Harvey Bradshaw Index (HBI) ≤4 points (14). The exclusion criteria were the following: symptomatic CD at enrolment with HBI ≥5 points or complications which included bowel obstruction and intraabdominal abscess; exclusive or predominant perianal CD; ileo- or colostomy; previous extended bowel resections; pregnancy; patients receiving steroids, nonsteroidal anti-inflammatory drugs, or antibiotics, and changes of the medication dose for immunosuppressants (azathioprine, 6-mercaptopurine, and methotrexate) or biologics (anti-TNF agents) within 3 months prior to enrolment. Data on socio-demographic and disease characteristics were collected at inclusion.

The enrolled patients were followed every 3 months for a 1-year period or less in the case of relapse. During the whole follow-up, the medication habits were kept track of so that no medication was added or changed in its dosage, except in case of relapse. The patients were instructed to communicate with the research coordinator if they developed symptoms suggesting exacerbation, to arrange a medical visit in order to confirm relapse, to have a blood sample taken, and to recover a stool sample from the previous day. Clinical relapse was defined by HBI ≥5 points associated with the increase of an objective marker of inflammation [serum ultrasensitive C-reactive protein (usCRP) above 4.8 mg/L and/or fecal calprotectin (fCal) above 250 µg/g stools]. Medical visits were carried out in 3-month intervals; upon relapse, CD activity was recorded (HBI), and a blood sample was collected for the measurement of serum usCRP, various CD4^+^ T cell subsets, and cytokines. The protocol was reviewed and approved by the Research and Ethics Committee of the Hospices Civils de Lyon, and written informed consent was obtained from each participant prior to study enrolment.

### Cell isolation and culture, flow cytometry acquisition, and analysis of CD4^+^ T cell subsets

Fresh blood samples were promptly transported to the laboratory for analysis. Heparinized venous blood samples were diluted 1:3 with phosphate-buffered saline (PBS), layered on a Ficoll-Hypaque density gradient, and then centrifuged for 30 min at 900 × *g*, and peripheral blood mononuclear cells (PBMCs) were collected from the interface. The PBMCs were suspended in RPMI 1640 culture medium supplemented with 100 U/ml penicillin, 100 µg/ml streptomycin, 2 mM L-glutamine, and 10% heat-inactivated fetal bovine serum and cultured in the presence or absence of phorbol-12-myristate-acetate (5 µg/ml; Sigma-Aldrich, Saint-Quentin Fallavier, France) and ionomycin (5 nM; Sigma-Aldrich) for 5 h in the presence of a protein transport inhibitor solution containing monensin (BD GolgiStop; BD Biosciences, San Diego, CA, USA) according to the manufacturer’s instructions. The incubator was set at 37°C under a 5% CO_2_ atmosphere. After 5 h of incubation, the cells were extensively washed with PBS and then surface-stained with Alexa Fluor 700-conjugated CD3 (clone UCHT1) and PeCy7-conjugated CD4 (clone SK3). Intracellular and intranuclear stainings were performed according to the manufacturer’s instructions (BD Biosciences). Briefly, the cells were permeabilized, fixed (BD^Ó^ Cytofix/Cytoperm Plus), and then stained with Alexa Fluor 647-conjugated FOXP3 (clone 236A/E7), PE-conjugated IL-17A (clone N49-653), and FITC-conjugated IFNγ. For each surface and intracellular staining, the relevant isotype control was used. All mAbs used for stainings were purchased from BD Pharmingen (Pont de Claix, France). Flow-Count Fluorospheres (Beckman Coulter, Brea, CA, USA) was used for the absolute count of cells. Immunostainings were acquired and analyzed by flow cytometry on a LSRII analyzer with the FACSDiva software (Becton Dickinson, San Jose, CA, USA), and analysis was performed using FlowJo software (Tree Star Inc., Ashland, OR, USA). The peripheral frequencies and the number of the following CD4^+^ T cell subsets were investigated: IL-17A^+^-producing T cells, IFNγ-producing T cells, FOXP3^+^-positive T cells, IFNγ^+^-producing FOXP3^+^ T cells, IL-17A-producing FOXP3^+^ T cells, and IFNγ- and IL-17A-producing T cells (**Supplementary Figure S1**). Th1 cells were identified as CD3^+^CD4^+^IFNγ^+^, Th17 cells as CD3^+^CD4^+^IL-17A^+^, and Tregs as CD3^+^CD4^+^FOXP3^+^ cells.

### Serum cytokine measurements

The ultrasensitive Erenna Immunoassay^®^ (Singulex, Alameda, CA, USA) was used to measure various cytokine levels in the serum (15). Erenna^®^ is a fluorescence immunoassay using nanobeads that allow the quantitation fluorescence of nanomolar concentrations of cytokines in serum samples. Briefly, cytokine-specific capture antibodies were pre-coated onto paramagnetic microparticles (MP). The MP, recombinant cytokine standards, and serum samples were pipetted into coated microplate wells. Cytokine binding to the captured antibody coated onto the MP was detected by incubation with a fluorescence-labeled detection antibody. Unbound antibody was eliminated by plate washes, and the MP were transferred to a clean plate. An elution buffer was then added and incubated. The elution buffer dissociated the MP-bound protein sandwich complexes, releasing the labeled antibodies. These antibodies were separated during transfer to a final microplate. The plate was loaded onto the Erenna^®^ System where the labeled molecules were detected and counted. The number of fluor-labeled detection antibodies counted was directly proportional to the amount of cytokines present in the sample when captured. The amounts of cytokines in unknown samples were interpolated from a standard curve. Prior to measurements and for each cytokine, all characteristics of dosage had been fully validated following the FDA guideline (15). The limits of quantification of the assays were 0.04 and 0.10 pg/ml for IL-6 and IL-17A, 0.30 pg/ml for TNFα, IFNγ, and IL-13, and 0.39, 0.98, 1.4, and 62.5 pg/ml, respectively, for IL-10, IL-8, IL-23, and TGFβ (16).

### Statistical analysis

The sample size was calculated based on comparable studies showing that CRP was a predictor of earlier relapse (17, 18). These studies included ≈100 CD patients, and 10–15% of patients were expected to not follow up. Statistical analyses were performed using both GraphPad Prism (La Jolla, CA, USA) and SAS 9.2 (Cary, NC, USA) software. Chi-square test was used to compare the patients’ characteristics. Results from numerical data were presented as mean, SEM, range, median, and 95% confidence interval (95%CI). Non-parametric Mann–Whitney test and Wilcoxon matched-pairs signed rank test were used to compare means appropriately. Results were considered statistically significant at 95% level of confidence (*p* < 0.05).

As our study focused on immunological parameters, the individual ability of each parameter to predict relapse was assessed using receiver operating characteristic (ROC) curves, allowing the definition of the best threshold associated with relapse in the subsequent months. In this analysis, when relapse occurred during the first month of the quarter, immunological data collected at the beginning of the previous quarter were chosen, *i*.*e*., a sample taken less than 4 months before the recording of the clinical relapse. In other cases, data collected at the beginning of the quarter were used, *i*.*e*., a sample taken between 1 and 3 months before relapse. Uni- and multivariate logistic regressions, including dichotomized variables defined by previously identified thresholds, were then performed to identify potential immunological predictive factors of relapse. Variables that achieved a *p <*0.1 value on univariate analysis were included in the multivariate analysis.

Multivariate logistic regressions were performed using binary factors from univariate analyses. The stepwise method was used with a significant level for entering and removing effects of 10%. *P*-values <0.10 were considered statistically significant. The odds ratios (ORs) were expressed with 95%CI. The model prediction was evaluated using the area under the curve from the ROC curve.

## Results

### Characteristics of the cohort of CD patients in remission at inclusion

**Figure 1:**
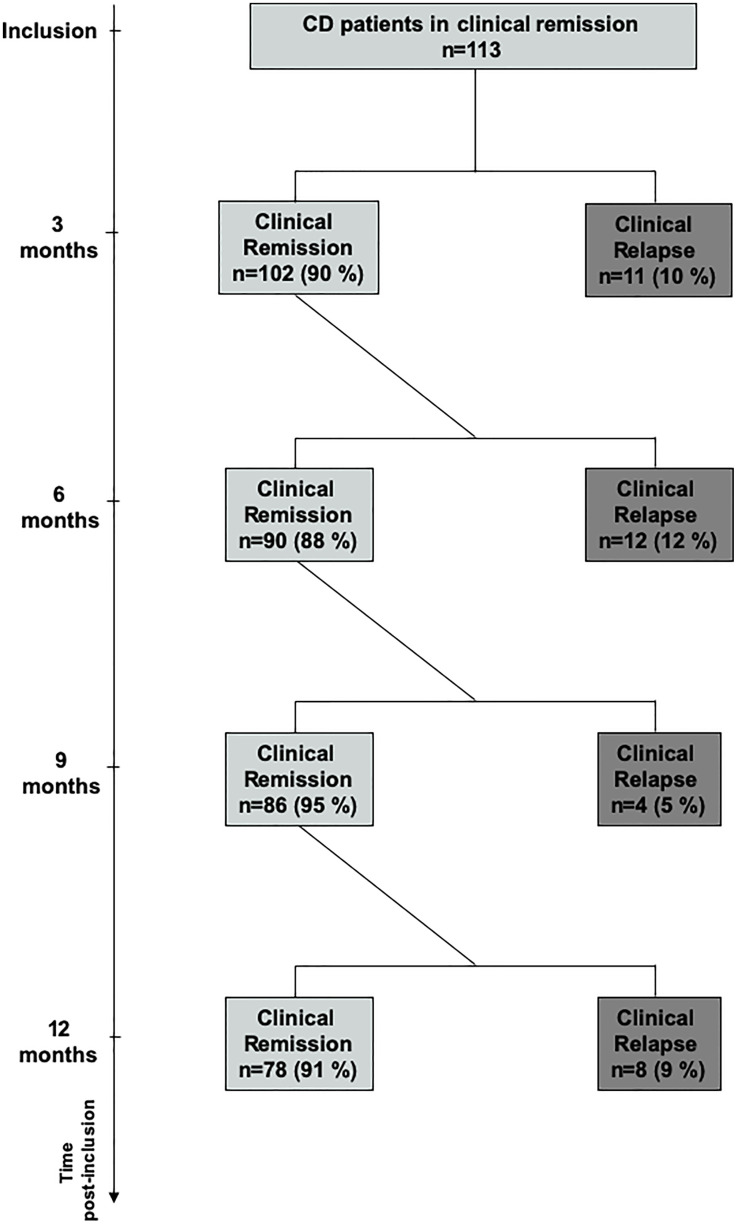
Flow chart of the cohort of Crohn’s disease patients.

A total of 113 CD patients were prospectively enrolled, and no patient was lost in the follow-ups. The flow chart of patients is given in **Figure 1**, and the baseline characteristics of the population are summarized in **Table 1**. The mean disease duration was 8.1 years, and 44% of patients had a prior history of intestinal resection. Moreover, 30% of patients were treated with a stable dose of immunosuppressants, and 50% were under anti-TNF therapy combined with immunosuppressants in 12% of cases. The mean time interval between enrolment and the last flares of CD was 2.4 years. Thirty-five patients (31%) experienced a relapse within the 1-year period of follow-up. In 31% of cases, relapses occurred during the second part of the first 3 months of follow-up, in 34% between 3 and 6 months, in 11% between 6 and 9 months, and in the remaining 23% between 9 and 12 months of follow-up (**Figure 1**). Patients who relapsed during the follow-up and patients who stayed in remission (non-relapsers) were similar in all baseline characteristics (**Table 1**). Half of the patients were under anti-TNF therapy.

**Table 1:** Baseline characteristics of the cohort of CD patients. P-value is calculated by comparing non-relapsers and relapsers.

Characteristics	All CD patients (n=113)	Non-relapsers (n=78)	Relapsers (n=35)	p
Age (yrs), mean (range)	38.3 (18.1-79.1)	38.0 (18.1-79.1)	39.3 (21.6-69.9)	0.72
Male gender, N (%)	44 (38.9)	33 (42.3)	11 (31.4)	0.27
Smokers, N (%)	36 (31.9)	26 (33.3)	10 (28.6)	0.62
Disease duration (yrs), mean (range)	8.1 (2.1-23.5)	7.4 (2.1-18.5)	8.9 (3.2-23.5)	0.45
Prior intestinal resection, N (%)	50 (44.2 %)	32 (41.0 %)	18 (51.4 %)	0.30
Time between enrolment and the last flare (yrs), mean (range)	2.4 (0.3-15.4)	2.5 (0.3-8.5)	2.0 (0.5-15.4)	0.24
Disease location*, N (%)				0.39
Ileal	23 (20)	15 (19)	8 (23)	
Colonic	12 (10)	6 (8)	6 (17)	
Ileocolonic	78 (69)	57 (73)	21 (60)	
Perianal	51 (45)	33 (42)	18 (51)	
Disease phenotype*, N (%)				0.29
Non-stricturing – Non-				
penetrating	35 (31)	22 (28)	13 (37)	
Stricturing	41 (36)	32 (41)	9 (26)	
Penetrating	37 (32)	24 (31)		
Treatment, N (%)				0.32
No	25 (22)	18 (23)	7 (20)	
Mesalamine	12 (11)	7 (9)	5 (14)	
Thiopurines	32 (28)	21 (27)	11 (31)	
Methotrexate	2 (2)	0 (0)	2 (6)	
Anti-TNFα**	56 (50)	38 (49)	18 (51)	

*According to the Montreal classification; ** anti-tumor necrosis factor alpha.

### Dynamics of peripheral CD4^+^ T helper cell subsets in CD patients in remission within 4 months prior to relapse and at the time of relapse

**Figure 2:**
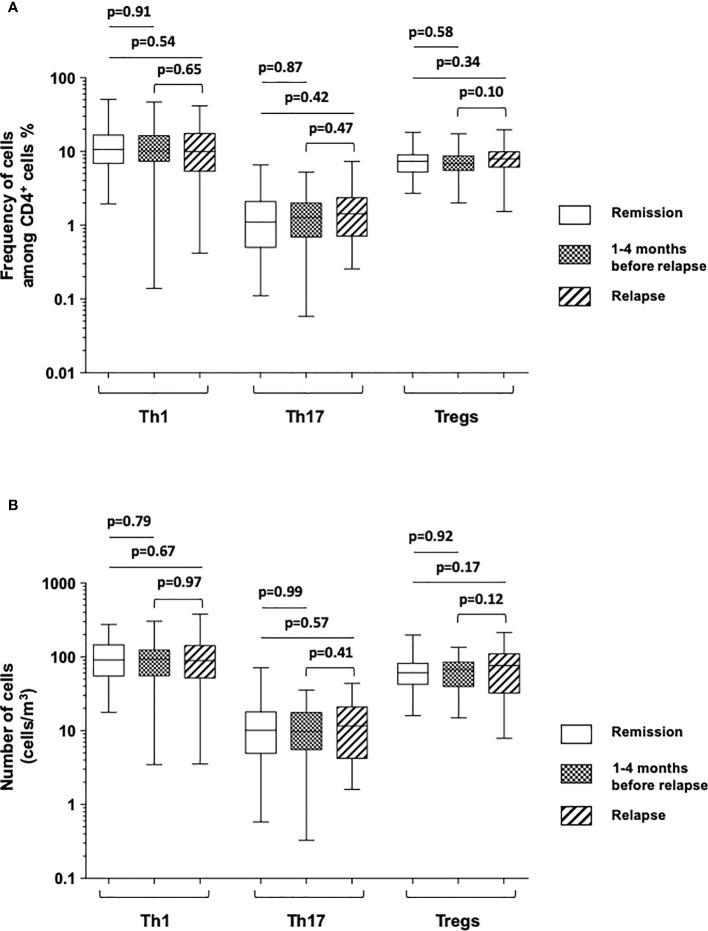
Comparison of the frequencies of cells among CD4^+^ T cells **(A)** and absolute number of cells [cells/mm (1)] **(B)** of Th1 (IFNγ-expressing CD4^+^ T cells), Th17 (IL-17A-expressing CD4^+^ T cells), and Tregs (FOXP3^+^-expressing CD4^+^ T cells) among Crohn’s disease patients who stayed in remission all throughout the follow-up (white), those who were in remission at the time of the measurement but who experienced a relapse during the following 1–4 months (boxed), and at the time of relapse in patients who relapsed (shaded). The box plots show the median, upper, and lower quartiles of the data; the whiskers indicate the 95% confidence interval of the values.

We firstly investigated the frequency and the number of Th1, Th17, and Treg cell subsets in peripheral blood and compared the prevalence of each CD4^+^ T cell subset at the time of relapse or within 4 months prior to relapse to that of patients who stayed in remission throughout the study period (*n* = 78) (**Figure 2** and **Table 2**). To precisely characterize these last patients, we used the data collected 3 months prior to the end of the study in patients still in remission at 3 months later.

**Table 2:** Frequencies and absolute numbers of CD4^+^ T helper cell subsets and concentration of serum cytokines (mean ± SEM).

	Remission	1-4 months before relapse	Relapse
Frequencies and absolute numbers of CD4^+^ T helper cell subsets (mean ± SEM)			
**Th1**			
Frequency (%)	12.80 **±** 1.13	12.58 **±** 1.51	11.67 **±** 1.47
Number (cells/mm^3^)	105.50 **±** 7.82	109.30 **±** 12.81	112.40 **±** 16.61
**Th17**			
Frequency (%)	1.46 **±** 0.16	1.50 **±** 0.20	1.68 **±** 0.23
Number (cells/mm^3^)	12.98 **±** 1.55	12.96 **±** 1.62	14.46 **±** 2.09
**Treg**			
Frequency (%)	7.52 **±** 0.38	7.16 **±** 0.54	8.17 **±** 0.58
Number (cells/mm^3^)	66.81 **±** 4.60	66.08 **±** 5.42	79.34 **±** 9.01
**IFNγ+FOXP3+**			
Frequency (%)	1.04 **±** 0.16	0.94 **±** 0.14	0.95 **±** 0.12
Number (cells/mm^3^)	8.05 **±** 0.74	7.73 **±** 0.91	8.98 **±** 1.39
**IL-17A+FOXP3+** Frequency (%)	0.17 **±** 0.02^*^	0.27 **±** 0.04^*^	0.19 **±** 0.04
Number (cells/mm^3^)	1.45 **±** 0.16^*^	2.44 **±** 0.39^*#^	1.32 **±** 0.20^#^
**IFNγ+IL-17A+**			
Frequency (%)	0.24 **±** 0.04	0.22 **±** 0.03	0.18 **±** 0.02
Number (cells/mm^3^)	1.88 **±** 0.25	1.83 **±** 0.23	1.56 **±** 0.22
Concentrations of serum cytokines (mean ± SEM)			
**IL-6**	0.99 **±** 0.16 pg/mL ^°^	1.29 **±** 0.25 pg/mL ^+^	2.28 **±** 0.38 pg/mL ^° +^
**IL-8**	6.77 **±** 0.99 pg/mL	6.21 **±** 0.84 pg/mL ^+^	7.93 **±** 1.12 pg/mL ^+^
**IL-17A**	0.59 **±** 0.15 pg/mL	0.46 **±** 0.09 pg/mL ^+^	0.80 **±** 0.18 pg/mL ^+^
**TNFα**	3.76 **±** 0.46 pg/mL	3.46 **±** 0.34 pg/mL	3.78 **±** 0.42 pg/mL
**IFNγ**	1.84 **±** 0.61 pg/mL	2.32 **±** 1.84 pg/mL	1.21 **±**0.36 pg/mL
**TGFβ**	16.60 **±** 1.83 ng/mL	20.85 **±** 2.46 ng/mL	16.99 **±** 2.29 ng/mL
**IL-23**	11.73 **±** 4.22 pg/mL	19.73 **±** 9.84 pg/mL	8.57 **±** 3.29 pg/mL
**IL-13**	3.58 **±** 0.81 pg/mL	6.88 **±** 2.79 pg/mL	4.10 **±** 1.60 pg/mL
**IL-10**	1.73 **±** 0.22 pg/mL	2.12 **±** 0.46 pg/mL	2.16 **±** 0.43 pg/mL

TGFβ is expressed in ng/mL.

*Remission vs relapse: p < 0.001.

#1-4 months vs relapse: p = 0.03.

^°^Remission vs relapse: p = 0.0007.

^+^1-4 months vs relapse: p < 0.004.

We failed to detect any significant difference when comparing the mean frequencies and numbers of circulating Th1, Th17, and Treg subsets between patients at the time of relapse and patients still in remission 3 months prior to the end of the study (**Figure 2** and **Table 2**). There was no difference likewise between these subsets when measured from 1 to 4 months before the occurrence of relapse and at the time of relapse in patients who relapsed (**Figure 2** and **Table 2**).

### Double-positive CD4^+^ T cell subsets with double-polarization CD4^+^IFNg^+^FOXP3^+^, CD4^+^IL-17A^+^FOXP3^+^, and CD4^+^IFNg^+^IL-17A^+^FOXP3- cells in CD patients in remission within 4 months prior to relapse and at the time of relapse

**Figure 3:**
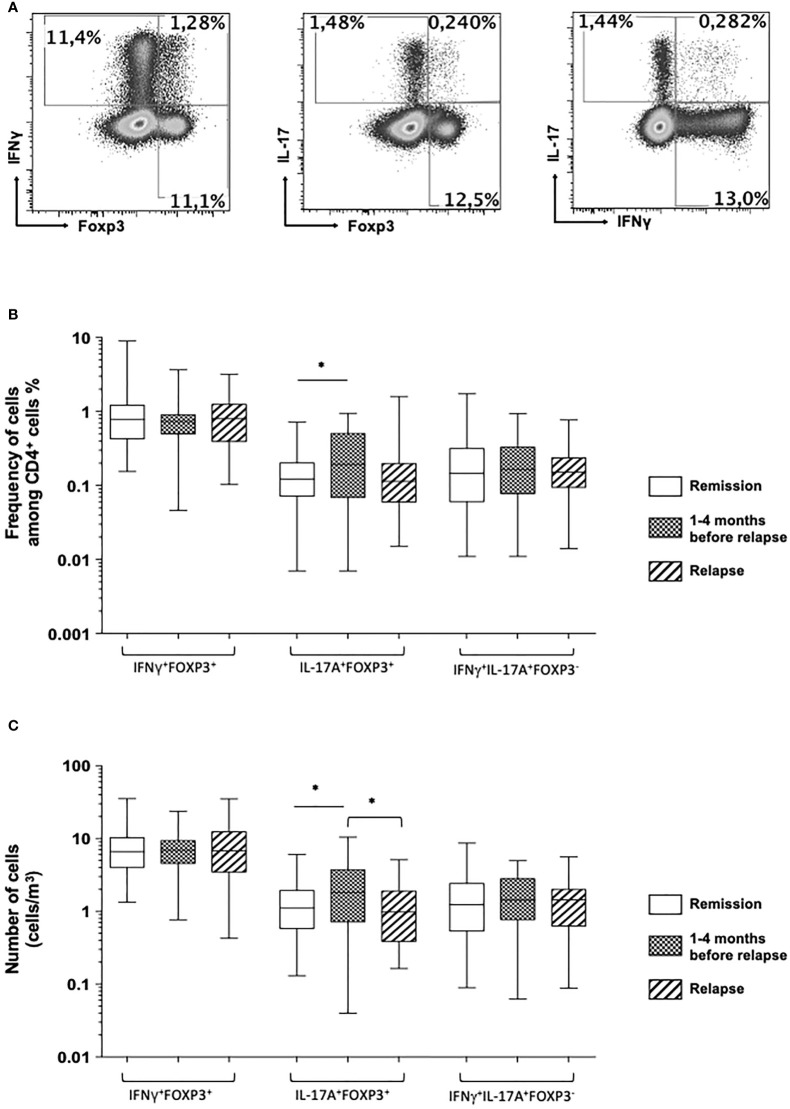
**(A)** Representative dot plot analysis of circulating double-positive FOXP3 and IFNγ CD4^+^ T cell subsets (left), FOXP3 and IL-17A CD4^+^ T cell subsets (middle), and double-expressing IL-17A and IFNγ CD4^+^ T cell subsets (right panel). Comparison of the frequencies **(B)** and absolute number **(C)** of the double-positive CD4^+^IFNγ^+^FOXP3^+^, CD4^+^IL-17A^+^FOXP3^+^, and CD4^+^IFNγ^+^IL-17A^+^FOXP3^-^ T cell subsets among Crohn’s disease patients who stayed in remission all throughout the follow-up (white), those who were in remission at the time of measurement but who experienced a relapse during the following 1–4 months (squared), and at the time of relapse in patients who relapsed (shaded). The box plots show the median, upper, and lower quartiles of the data; the whiskers indicate the 95% confidence interval of the values (*p< 0.05).

Next, we investigated whether circulating double-positive CD4^+^ T cell subsets co-expressing IFNγ and FOXP3, IL-17A and FOXP3, or IFNγ and IL-17A were detectable in patients and whether the proportions and numbers of these subsets had changed before and at the time of relapse (**Figure 3** and **Table 2**). Although their mean frequencies among the whole circulating CD4^+^ T cells were relatively weak, each of these circulating double-positive CD4^+^IFNγ^+^FOXP3^+^, CD4^+^IL-17A^+^FOXP3^+^, and CD4^+^IFNγ^+^IL-17A^+^FOXP3^-^ T cell subsets was detectable in patients in remission and before and at the time of relapse (**Figure 3** and **Table 2**).

When comparing the mean frequencies and numbers of circulating double-positive CD4^+^ T cell subsets co-expressing IFNγ and FOXP3, IL-17A and FOXP3, or IFNγ and IL-17A, we failed to detect any significant difference between patients who stayed in remission and at the time of relapse for those who relapsed. Compared with patients who stayed in remission, there was a significant increase in the mean frequency and absolute number of circulating double-positive IL-17A-producing FOXP3^+^ CD4^+^ T cells between 1 and 4 months before the occurrence of a relapse (0.17 ± 0.02 *vs*. 0.27 ± 0.04%, *p* = 0.01, and 1.45 ± 0.16 *vs*. 2.44 ± 0.39 cells/mm (1), *p* = 0.008) (**Figure 3** and **Table 2**). The samples taken from patients between 1 and 4 months prior to their relapse strikingly exhibited an increase in the frequency and absolute number of circulating double-positive IL-17A-producing FOXP3^+^ CD4^+^ T cells. The relapsed sample levels were similar to those measured in patients who stayed in remission (**Figure 3** and **Table 2**).

### Changes in usCRP and in a wide panel of serum cytokines in patients with CD in remission within 4 months prior to relapse and at the time of relapse

As expected, the mean usCRP was significantly increased in relapsers at the time of relapse compared with patients who stayed in remission (16.7 ± 3.8 vs. 4.4 ± 0.8 mg/L, *p* = 0.0001). This increase was already noted between 1 and 4 months before the occurrence of a relapse (10.1 ± 2.6 vs. 4.4 ± 0.8 mg/L, *p* = 0.01).

**Figure 4:**
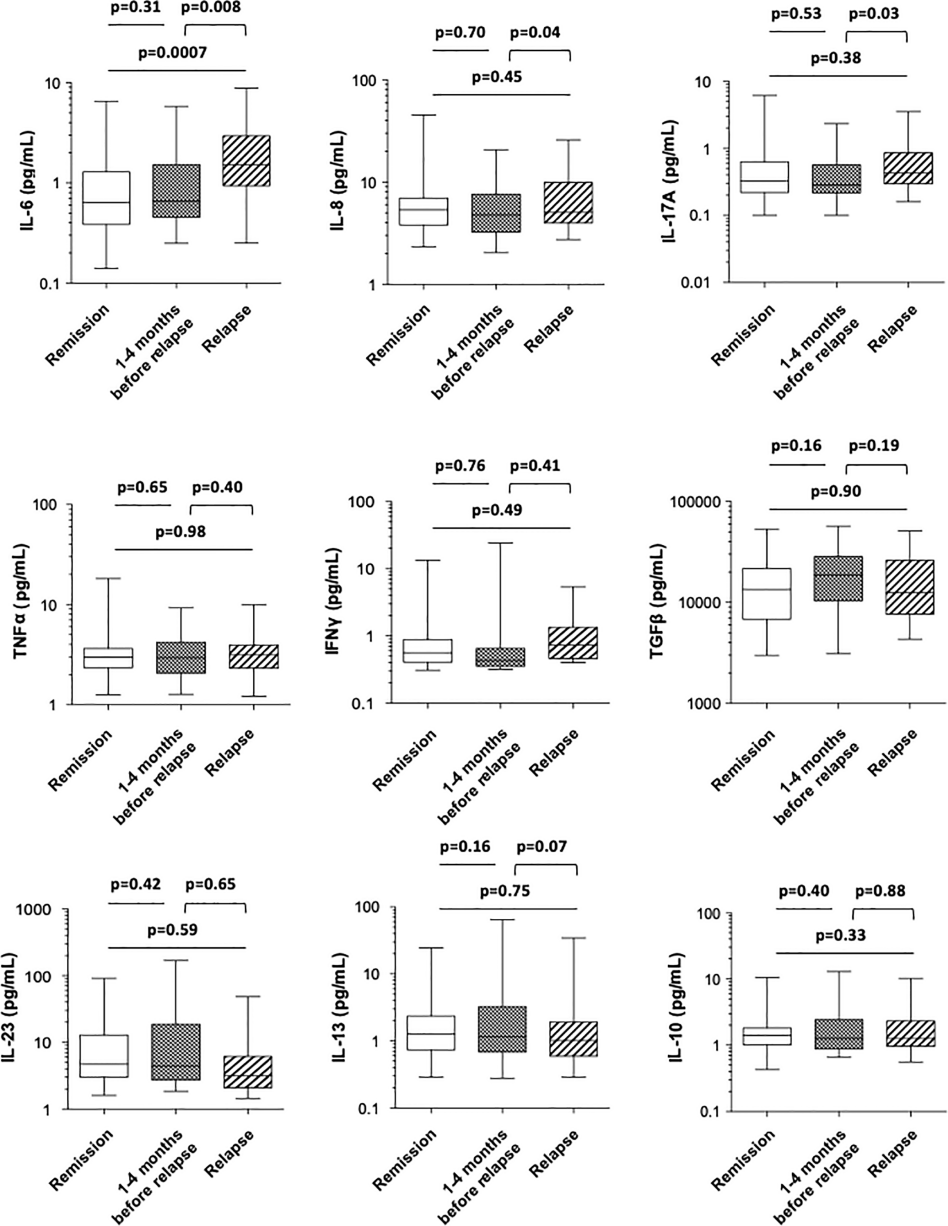
Comparison of the blood concentrations of various cytokines (IL-6, IL-8, IL-17A, TNFα, IFNγ, TGFβ, IL-23, IL-13, and IL-10) among Crohn’s disease patients who stayed in remission all throughout the follow-up (white), those who were in remission at the time of measurement but who experienced a relapse during the following 1–4 months (boxed) and at the time of relapse in patients who relapsed (shaded). The box plots show the median, upper, and lower quartiles of the data; the whiskers indicate the 95% confidence interval of the values.

When comparing the levels of a large panel of cytokines in CD patients who stayed in remission with those of patients at the time of relapse, we found that only the mean level of the proinflammatory cytokine IL-6 was significantly higher under clinical relapse (0.99 ± 0.16 *vs*. 2.28 ± 0.38 pg/ml, *p* = 0.0007) (**Figure 4** and **Table 2**). The mean concentrations of serum TNFα did not differ between patients in remission and those who relapsed (3.76 ± 0.46 *vs*. 3.78 ± 0.42 pg/ml, respectively; *p* = 0.98), while they were not affected by anti-TNF therapy. Within 4 months prior to the occurrence of relapse, there was no apparent difference in the levels of cytokines compared with patients who stayed in remission (**Figure 4** and **Table 2**). Finally, upon comparing the levels of various cytokines measured between 1 and 4 months prior to relapse with those measured subsequently at the time of relapse in patients who relapsed, we found that the mean levels of proinflammatory cytokines, including IL-6, IL-8, and IL-17A, were significantly higher at the time of relapse (1.29 ± 0.25 *vs*. 2.28 ± 0.38 pg/ml for IL-6, *p* = 0.008; 6.21 ± 0.84 *vs* 7.93 ± 1.12 pg/ml for IL-8, *p* = 0.04; and 0.46 ± 0.09 *vs*. 0.80 ± 0.18 pg/ml for IL-17A, *p* = 0.03) (**Figure 4** and **Table 2**).

### Predictors of CD relapse within 4 months prior to relapse

**Figure 5:**
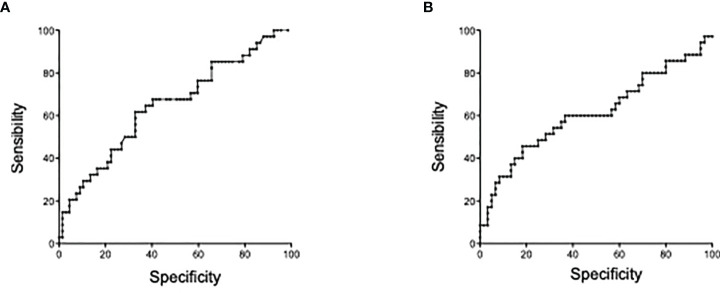
Predictors of Crohn’s disease relapse within 4 months prior to relapse. **(A)** Receiver operating characteristic (ROC) curve of comparison between mCRP and risk of relapse at 4 months. **(B)** ROC curve of IL-17+FOXP3+CD4 T cell numbers and risk of relapse at 4 months.

The optimal thresholds of immunological markers to predict clinical relapse were determined by ROC curves. On the univariate analysis by logistic regression (**Table 3**), usCRP ≥3.44 mg/L, absolute numbers of circulating double positive IL-17A producing FOXP3^+^ CD4^+^ T cells ≥1.4 cells/mm (1), and IL-10 concentrations less than 1.34 pg/ml were associated with a further relapse and were included in the multivariate analysis. In the multivariate analysis, a statistically significant association was detected between usCRP levels ≥3.44 mg/L and the risk of a further relapse (OR = 6.13; 95%CI = 2.20–17.09; *p* < 0.001) (**Figure 5**). Another significant and independent predictor of further relapse was the number of CD4^+^ double-positive FOXP3^+^IL-17A^+^ in peripheral blood since a high number [≥1.4 cells/mm (1)] was associated with a higher risk of relapse (OR = 2.81; 95%CI =1.13–7.04; *p* = 0.03) (**Figure 5**). Moreover, there was no correlation between CD4^+^ double-positive FOXP3^+^IL-17A^+^ associated with relapse and the increased levels of IL-17A. Among the other CD4^+^ T helper cell subsets and cytokines that were assessed in the multivariate model, none of them was associated with a high risk of clinical relapse.

**Table 3:** Univariate and multivariate analysis of biological parameters associated with the risk of further relapse.

Parameters	Univariate analysis		Multivariate analysis	
	OR (95% CI)	p value	OR (95% CI)	p value
**hsCRP ≥ 3.44 mg/L**	5.47 (2.12 - 14.15)	p<0.001	6.13 (2.20 - 17.09)	p<0.001
**Th1**				
Frequency (%)	1.01 (0.97 - 1.06)	p=0.64		
Number (cells/mm^3^)	1.00 (1.00 - 1.01)	p=0.45		
**Th17**				
Frequency (%)	1.12 (0.80 - 1.58)	p=0.50		
Number (cells/mm^3^)	1.00 (0.97 - 1.04)	p=0.78		
**Treg**				
Frequency (%)	0.99 (0.86 - 1.14)	p=0.87		
Number (cells/mm^3^)	1.00 (0.99 - 1.01)	p=0.86		
**IFNγ+FOXP3+**				
Frequency (%)	1.11 (0.66 - 1.87)	p=0.70		
Number (cells/mm^3^)	1.02 (0.93 - 1.11)	p=0.71		
**IL-17A+FOXP3+**				
Frequency (%)	1.38 (0.55 - 3.46)	p=0.49		
Number (cells/mm^3^)	2.89 (1.24 - 6.77)	p=0.01	2.81 (1.13 - 7.04)	p=0.03
**IFNγ+IL-17A+FOXP3^-^**				
Frequency (%)	1.16 (0.23 - 5.99)	p=0.86		
Number (cells/mm^3^)	1.01 (0.81 - 1.26)	p=0.92		
**IL-6** (pg/mL)	2.02 (0.75 - 5.47)	p=0.17		
**IL-8** (pg/mL)	1.62 (0.62 - 4.23)	p=0.32		
**IL-17A** (pg/mL)	1.67 (0.60 - 4.60)	p=0.31		
**TNFα** (pg/mL)	0.76 (0.29 - 1.97)	p=0.57		
**IFNγ** (pg/mL)	0.48 (0.13 - 1.81)	p=0.28		
**TGFβ** (pg/mL)	1.51 (0.58 - 3.89)	p=0.40		
**IL-23** (pg/mL)	1.46 (0.39 - 5.40)	p=0.57		
**IL-13** (pg/mL)	1.45 (0.54 - 3.85)	p=0.46		
**IL-10** (pg/mL)	2.57 (0.95 - 6.96)	p=0.06		

## Discussion

In the present study, we studied changes in CD4^+^ T cells (Th1, Th17, and Treg) and double-expressing IL-17A-IFNγ, IL-17A-FOXP3, and IFNγ-FOXP3 CD4^+^ T cell subsets in a large cohort of clinically well-characterized CD patients. We completed the analysis of circulating T cell subsets with the concomitant measurement of most of the serum cytokines using an ultrasensitive tool capable of detecting very low concentrations of the proteome contrary to common assays (ELISA or Luminex). Our longitudinal design allowed the systematic monitoring of various immunological parameters prior to CD relapse. We defined the relapse according to the widely used HBI in clinical practice in association with the objective parameters of mucosal inflammation such as usCRP and/or fCal.

Contrary to patients who stayed in remission, neither CD4^+^ T cell subsets or cytokines were different at the time of relapse, with the exception of IL-6. Within 1 to 4 months that preceded a relapse, the number of circulating double-positive IL-17A-producing FOXP3^+^ CD4^+^ T cells increased and, with a cutoff value ≥1.4 cells/mm (1), was a predictive measurement of a further relapse. In addition, usCRP increased not only at the time of relapse but also in the preceding months and was predictive of earlier relapses at a cutoff value ≥3.44 mg/L. Interestingly, two inflammatory markers, IL8 and IL-17A, were significantly higher at the time of relapse. This could be explained by the fact that intestinal inflammation in Crohn’s disease starts in the mucosal secretion of IL-8 and IL-17A, which can be found in significant amounts in blood as inflammation progresses with time. With respect to usCRP assay, as a more sensitive way of titrating mucosal inflammation than the standard CRP assay, our present data are in line with previous studies supporting CRP as a strong predictor of relapse (17–21). Whether nanomolar CRP level titrated by usCRP assay Arena^®^ allows for the earlier detection of CRP during the relapsing episode warrants further studies. CRP hepatic production is regulated by proinflammatory cytokines, which are predominantly IL-6 and are usually correlated. CRP is also expressed by adipocytes in mesenteric fat pads in patients with CD (22). In our study, nanomolar quantities of CRP, as titrated by the usCRP Arena^®^ assay, increased prior to relapse, but not circulating IL-6, a dissociation already evidenced by Bitton et al. (18). The poor correlation between circulating CRP and IL-6 levels could be due to confounding factors that might influence the production and/or detection of free CRP and IL6 in the serum.

Our study confirms and extends the notion that circulating T cells co-expressing IL-17A and IFNγ, previously reported both in mouse and human peripheral and mucosal compartments (23), represent a decreased subset of functional T cells appearing as markers several months before IBD relapse episodes. Moreover, it highlights the weak but detectable number of CD4^+^ T cells co-expressing FOXP3 and IL17A or IFNγ. While FOXP3 is a transcription factor with a key role for Treg differentiation and suppressive function in mice, lymphocytes expressing FOXP3 in humans can harbor a suppressive function as classical Tregs depending on the level of FOXP3 transcripts/protein as well as the co-production by the same cells (or within the gut microenvironment) by pro-inflammatory mediators; could represent cells that, due to pro-inflammatory microenvironmental cues, adopt a more adapted phenotype to either attempt to jugulate inflammation or, on the contrary, cells that might, if chronic inflammation is severe, become themselves effector cells with deleterious function. It could also be possible that the presence of such non-classical Tregs co-producing IFNγ or IL17 might reflect treatment failure and resistance to anti-TNFα. Along this line, our findings indicate a significant rise in the FOXP3^+^ IL17A^+^ CD4^+^ T cell subset during the preceding 1 to 4 months of the occurrence of relapse in CD patients in the remission phase, thus suggesting that this subset could be a potentially relevant pathophysiological indicator of the complex and multifactorial CD relapse episodes.

The origin of these double-positive IL-17A-producing FOXP3^+^ CD4^+^ T cells still remains elusive. Importantly, their presence in disease situations supports the concept of T cell plasticity of Tregs geared towards either a more Th1 pro-inflammatory/effector phenotype or acquiring a regulatory function in an ongoing inflammatory context. We cannot exclude, on the contrary, that Th17 cells could also acquire the expression of FOXP3, as the presence of T cells with low levels of FOXP3 is a general feature of early activated T cells in human which coincides with the defective suppressive function of FOXP3 CD4^+^ Tregs, supporting a possible conversion of Tregs into Th17 cells. Our findings illustrate Treg cell plasticity during the longitudinal time-dependent development of inflammatory flares in IBD, thus nicely complementing previous studies of increased prevalence of FOXP3^+^IL17A^+^ cells in active IBD patients’ serum (12).

Treg cells were shown to co-express IL-17A and to be able to modulate their suppressive functions when exposed to microenvironmental influences (11). Treg and Th17 cells were derived from the same cell lineage, and their differentiation is dependent on the presence of the canonical immunomodulatory cytokine TGFβ in the microenvironment, especially in the intestine (9). Our study though did not reveal any relationship between the levels of serum TGFβ and the frequencies or numbers of circulating Treg cells and Th17 CD4^+^ T cell subsets, including the double-positive IL-17A-producing FOXP3^+^ CD4^+^ T cells. Interestingly, a very low level of IL-10, which has anti-inflammatory effects, was detected in the serum by the ultrasensitive Erenna^®^-based technology and tended, in the univariate analysis, to be a factor associated with earlier relapse in CD.

To detect and monitor the levels of a wide panel of circulating cytokines involved in the pathogeny of CD, we used the ultrasensitive Erenna^®^ Immunoassay that is able to detect femtogram/milliliter levels of cytokines, *i*.*e*., a 1,000-fold improvement in sensitivity over other assays. This digital ELISA uses a well-calibrated assay reagent format coupled with the Erenna^®^ instrument that uses single-photon digital capture of the assay signal to achieve the detection of proteins that were undetectable using standard methods. To our knowledge, our study was the first to document the use of this ultrasensitive method to monitor the cytokine levels in IBD. Using this ultrasensitive method, we did not detect any increase of any serum cytokine in the months preceding CD relapse, which disagrees with some (24–26) but not all (27) previous studies, supporting that a high serum concentration of IL-6 was predictive of relapse. In contrast, when compared with the period prior to relapse, we found that IL-6 concentrations significantly increased at the time of relapse, which confirms previous studies (28, 29). Alternatively, we identified a rise in circulating IL-8 and IL-17A at the time of relapse, which was not previously documented in CD.

We emphasize that the evolution of numbers and phenotype of immune cells in IBD blood might not necessarily mirror cell phenotypes within mucosal sites, and thus the functional phenotype of CD4^+^ T helper cell subsets should be explored whenever possible in intestinal mucosal tissue samples as well as in blood samples. Importantly, whether these double-positive IL17A^+^FOXP3^+^ T cell subset harbors an immunoregulatory/suppressive function or, alternatively, might even convert into pro-inflammatory/pathogenic effectors during the pathophysiology of IBD remains an open question. Along these lines, we previously documented using *in vivo* approaches and transfer models with wild transgenic and reporter mice that even during colitis T cells can convert into Foxp3^+^ T cells within the colon mucosa, thus retaining an *in vivo* suppressive function alleviating colitis (10). Besides this, we were the first to identify in an original model of DTH-mediated colitis to hapten-modified self that cytolytic CD8^+^ T cells could initiate the inflammatory process in T cell-mediated colitis and in Balb/C mice as well as in MHC class II knock out (*i*.*e*., CD4^+^ T cell-deficient) B6 mice (30). Moreover, in humans, in the setting of post-operative endoscopic relapse of IBD, we have previously shown that there were cytotoxic CD8+ T cells that occur at the ileo-colonic anastomosis site at 3 to 4 months prior to clinical relapse (31). It may thus be proposed that the present study further reveals a small but detectable rise in circulating IL-17A-producing FOXP3^+^ CD4^+^ T cell subset that was associated with the occurrence of a CD relapse within 4 months, supporting the contribution of this subset along with CD8 cytotoxic T cells as key pathophysiologic markers of a preclinical relapse that thus appear as potential diagnostic and therapeutic targets in IBD. It is thus possible to speculate, from our mouse and human data on IBD settings and Treg plasticity, that Foxp3+IL17A+ T cells are recruited from the blood into the inflamed gut mucosa later at the time of relapse, explaining its decrease in the blood, and/or that these cells can further differentiate into Th17A^+^ cells upon downregulation of FOXP3 expression; thus they might represent “ex-Treg”, subverted and unable to control inflammation, or even depending on the pro-inflammatory microenvironment and the individual’s natural history of IBD, changes in their microbiota might acquire pro-inflammatory helper/effector properties, thus explaining the difficulty and peculiarity of anti-biotherapies.

## Conclusion

In conclusion, these findings suggest that, despite their rareness and frequency in peripheral blood in comparison to other more classical CD4^+^ T helper cells, IL-17A-producing FOXP3^+^ CD4^+^ T cell subsets represent a useful pathophysiological marker of initial infra-clinical stages of CD relapse and a therapeutic target in CD.

## Data availability statement

The raw data supporting the conclusions of this article will be made available by the authors without undue reservation.

## Ethics statement

The studies involving human participants were reviewed and approved by the Research and Ethics Committee of the Hospices Civils de Lyon. The patients/participants provided their written informed consent to participate in this study.

## Author contributions

GB, SN, BF, and DK designed the study. RD-L, TA, KR, CM, CJ, RJ, and SL performed the research. RD-L, GB, XR, AR, CV, and AF analyzed and interpreted the data. RD-L, BF, SN, GB, DK, and MF wrote the manuscript with critical input from J-BL, AB, and XR. All authors contributed to the article and approved the submitted version.

## Funding

This study was supported by PHRC régional (Programme Hospitalier de Recherche Clinique) FUI (Fonds Unique Interministériel).

## Acknowledgments

We are grateful to the Cytometry Core Facility of the UMR5308 for help with the FACS analysis. We also acknowledge the Fondation pour la Recherche Médicale (FRM). We would like to thank Joseph Josephides for critically reading the manuscript.

## Conflict of interest

CJ and RJ were employed by Advanced Bioscience Laboratories (ABL). SL was employed by Indicia. AF was employed by TxCell.

The remaining authors declare that the research was conducted in the absence of any commercial or financial relationships that could be construed as a potential conflict of interest.

## Publisher’s note

All claims expressed in this article are solely those of the authors and do not necessarily represent those of their affiliated organizations, or those of the publisher, the editors and the reviewers. Any product that may be evaluated in this article, or claim that may be made by its manufacturer, is not guaranteed or endorsed by the publisher.

## Supplementary material

Supplementary Figure 1 Gating strategy. Among the total leucocytes, doublets cells, dead cells and debris were excluded based on SSC or FSC characteristics. Cell population were gating on CD3^+^ cells. CD4^+^ cells were gated on CD3^+^. IL17A, IFNγ and Foxp3 were evaluated among CD4^+^ populations **(A)**. Then Among FOXP3+ cells, IL17A and IFNγ positive cells were gating **(B)**. Among CD3+CD4+, IL17A^+^FOXP3^+^, FOXP3^+^IFNγ+ or IL17A+IFNγ+ cells were analyzed **(C)**.
