# Supplementary figures and images for: Relationships of circulating CD4+ T cell subsets and cytokines with the risk of relapse in patients with Crohn’s disease
Source: Front Immunol. 2022 Nov 4;13:864353. doi: 10.3389/fimmu.2022.864353 (PMC9674020; doi:10.3389/fimmu.2022.864353)

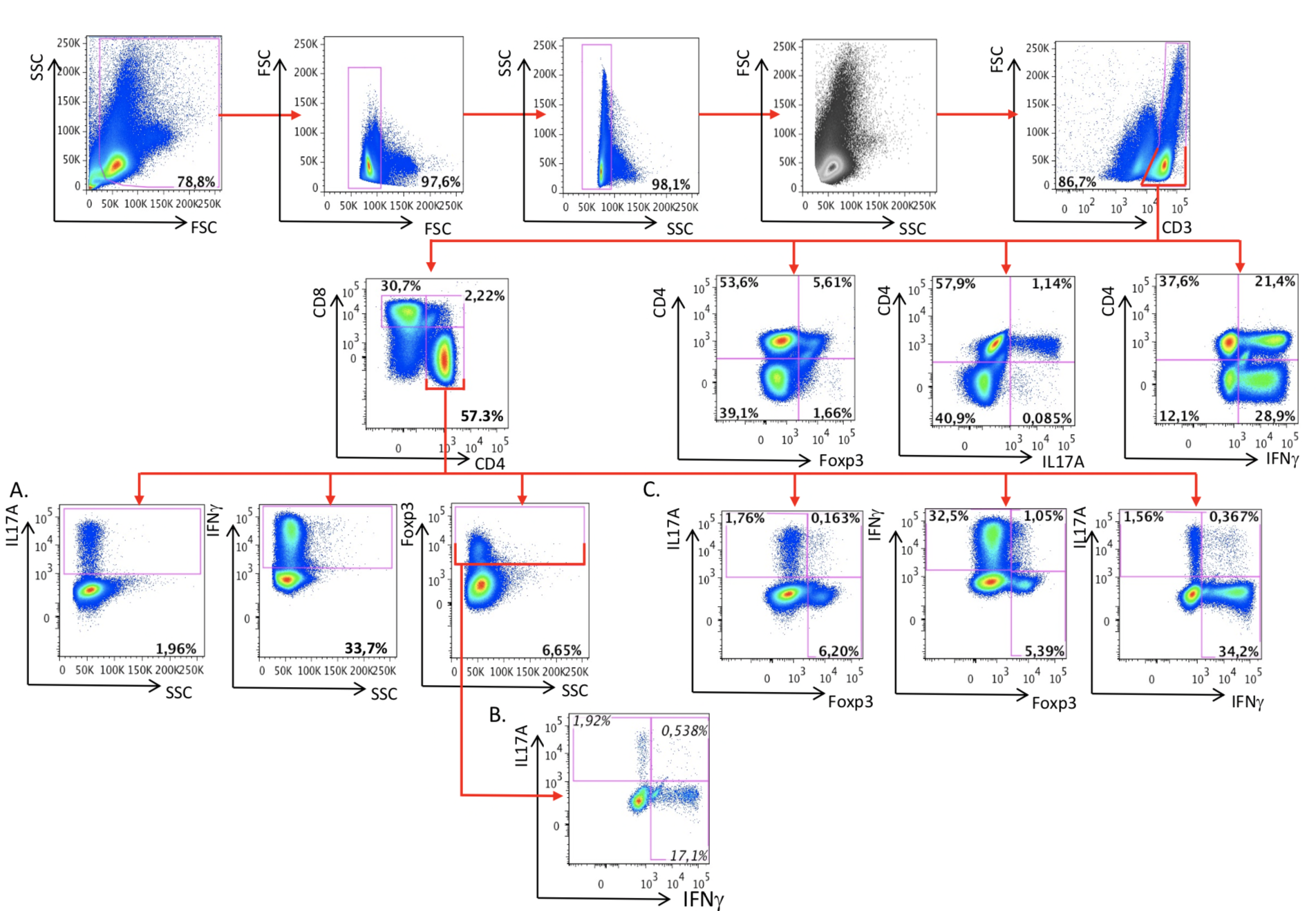

Supplement: Supplementary Figure 1 — Gating strategy. Among the total leucocytes, doublets cells, dead cells and debris were excluded based on SSC or FSC characteristics. Cell population were gating on CD3+ cells. CD4+ cells were gated on CD3+. IL17A, IFNγ and Foxp3 were evaluated among CD4+ populations (A). Then Among FOXP3+ cells, IL17A and IFNγ positive cells were gating (B). Among CD3+CD4+, IL17A+FOXP3+, FOXP3+IFNγ+ or IL17A+IFNγ+ cells were analyzed (C). [file Image_1.tiff]
